# Supplementary material for: Complexome profiling of the Chlamydomonas psb28 mutant reveals TEF5 as an early PSII assembly factor
Source: Plant Cell. 2025 Mar 17;37(6):koaf055. doi: 10.1093/plcell/koaf055 (PMC12164590; doi:10.1093/plcell/koaf055)
Supplement: koaf055_Supplementary_Data [file koaf055_supplementary_data.zip › psb28_interactive.zip!/plots/intensity/qualityPlots/peptides/ASA8_Cre09.g402300.t1.2.html]

### Cre09.g402300.t1.2

Trivial name: ASA8  
SeqCoverage [median]: 65.20  
Mass: 10.02 kDa  
  
 

| Sequence | MissedCleavage | Mass [Da] | wtIntensity | muIntensity | Score |
| --- | --- | --- | --- | --- | --- |
| AYDEAVLAGK | 0 | 1035.52 | 63253.20 | 73428.79 | 219.51 |
| AYDEAVLAGKQPFTAGGH | 1 | 1830.89 | 10704.03 | 34116.70 | 130.39 |
| DILRPPPTGFMPENVAHPYQK | 1 | 2406.22 | 10842.54 | 65870.90 | 135.81 |
| KAYDEAVLAGK | 1 | 1163.62 | 3400.15 | 4640.34 | 122.51 |
| KAYDEAVLAGKQPFTAGGH | 2 | 1958.98 | 0.00 | 781.86 | 41.41 |
| KKAYDEAVLAGK | 2 | 1291.71 | 1804.08 | 4064.57 | 101.89 |
| QPFTAGGH | 0 | 813.38 | 17613.02 | 21187.83 | 116.00 |
| SFYTYATK | 0 | 979.47 | 34643.17 | 44886.53 | 131.03 |
| SFYTYATKK | 1 | 1107.56 | 2334.79 | 3269.83 | 102.62 |
| TLGEAYLK | 0 | 893.49 | 2463.40 | 8480.97 | 96.19 |
|  |  |  | 147058.4 | 260728.3 |  |
